# Ordered Mesoporous Boron Carbon Nitrides with Tunable Mesopore Nanoarchitectonics for Energy Storage and CO_2_ Adsorption Properties

**DOI:** 10.1002/advs.202105603

**Published:** 2022-04-05

**Authors:** CI Sathish, Gopalakrishnan Kothandam, Premkumar Selvarajan, Zhihao Lei, Jangmee Lee, Jiangtao Qu, Ala'a H. Al‐Muhtaseb, Xiaojiang Yu, Mark B. H. Breese, Rongkun Zheng, Jiabao Yi, Ajayan Vinu

**Affiliations:** ^1^ Global Innovative Centre for Advanced Nanomaterials (GICAN) College of Engineering Science and Environment The University of Newcastle Callaghan NSW 2308 Australia; ^2^ School of Physics The University of Sydney Sydney New South Wales 2006 Australia; ^3^ Department of Petroleum and Chemical Engineering College of Engineering Sultan Qaboos University Muscat 33 Oman; ^4^ Singapore Synchrotron Light Source National University of Singapore Singapore 117603 Singapore; ^5^ Department of Physics National University of Singapore Singapore 117542 Singapore

**Keywords:** CO_2_ capture, mesoporous, sodium‐ion battery, specific capacitance

## Abstract

Porous boron carbon nitride (BCN) is one of the exciting systems with unique electrochemical and adsorption properties. However, the synthesis of low‐cost and porous BCN with tunable porosity is challenging, limiting its full potential in a variety of applications. Herein, the preparation of well‐defined mesoporous boron carbon nitride (MBCN) with high specific surface area, tunable pores, and nitrogen contents is demonstrated through a simple integration of chemical polymerization of readily available sucrose and borane ammonia complex (BAC) through the nano‐hard‐templating approach. The bimodal pores are introduced in MBCN by controlling the self‐organization of BAC and sucrose molecules within the nanochannels of the template. It is found that the optimized sample shows a high specific capacitance (296 F g^−1^ at 0.5 A g^−1^), large specific capacity for sodium‐ion battery (349 mAg h^−1^ at 50 mAh g^−1^), and excellent CO_2_ adsorption capacity (27.14 mmol g^−1^ at 30 bar). Density functional theory calculations demonstrate that different adsorption sites (B—C, B—N, C—N, and C—C) and the large specific surface area strongly support the high adsorption capacity. This finding offers an innovative breakthrough in the design and development of MBCN nanostructures for energy storage and carbon capture applications.

## Graphical abstract

A new class of mesoporous boron carbon nitrides exhibiting a rod‐like morphology with well‐defined pores are designed through a simple chemical polymerization and nanotemplating technology that proves its efficiency in multiple applications as energy storage devices and adsorption.

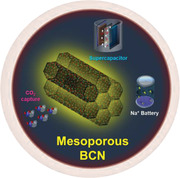

## 1 Introduction

Graphene‐based materials have been receiving a lot of attention in recent years owing to their outstanding thermal, electrical, and electronic properties such as ballistic transport, high thermal conductivity, and better electrical conductivity compared to metals.^[^ ^1^, ^2^ ^]^ Even though the gapless nature of graphene limits its applications in the semiconductor industries and many other areas, such as electrocatalysis, theoretical calculations predicted that the bandgap of graphene could be opened and tuned by doping with heteroatoms such as boron and nitrogen.^[^ ^3^ ^]^ Generally, graphene doped with nitrogen behaves as an n‐type semiconductor,^[^ ^4^ ^]^ whereas boron‐doped graphene shows a p‐type behavior.^[^ ^5^ ^]^ Moreover, nitrogen or boron doping can induce active sites, leading to excellent electrochemical properties for various applications, such as supercapacitors, batteries, and carbon dioxide capture.^[^ ^6^, ^7^ ^]^ In addition, reports have shown that when N and B are codoped in the graphene lattice, a cumulative effect for electrochemical properties from both elements is achieved.^[^ ^8^ ^]^

Boron carbon nitrides (BCNs) are an important class of materials with a unique band structure containing graphene, boron nitride along with BCN ring which offers an excellent platform for adsorption, photocatalytic, electrocatalytic, and photoredox applications.^[^ ^9^ ^]^ Unfortunately, BCN without any porous or optimized band structure is unavailing for energy storage or adsorption. Although there are numerous developments on controlling the porous structure and electronic properties of the BCN materials for various applications,^[^ ^10^ ^]^ it still suffers from low structural order and surface area, poor electronic conductivity, and high cost of production due to expensive precursors. Therefore, the design and development of ordered BCN with controlled porous structure and tunable conductivity are critical for maximizing this fascinating material's potential in the field of energy storage, adsorption, removal of complex chemical contaminants, and sensing.^[^ ^6^, ^11^, ^12^, ^13^ ^]^

Creating ordered mesopores in the nanomaterials is the key to open many hidden features of a particular material.^[^ ^14^ ^]^ Activation strategy has been adopted to introduce nanoporosity in BCN. Although this approach was successful in generating a high specific surface area, the lack of ordered pores limits its performance in the adsorption of CO_2_ and methane.^[^ ^12^, ^15^, ^16^ ^]^ The activation approach creates a lot of hidden meso‐ or micropores, which may not be accessible for the reactant or adsorbate molecules, significantly affecting its performance in adsorption and separation. Hard‐templating strategy is one of the unique strategies for synthesizing ordered mesoporous BCN materials. Our group previously reported an elemental substitution technique to create nanoporosity in BN and BCN nanostructures using mesoporous carbon as the template.^[^ ^16^ ^]^ However, this method proceeded BCN with disordered porous structure and poor specific surface area and required ultrahigh temperature, which increases the production cost of BCN. Hence, it is a challenge to fabricate ordered BCN with low cost and tunable pore sizes and conductivity for sodium‐ion batteries, supercapacitors, and CO_2_ adsorption. In this communication, we show a facile method to synthesize highly ordered mesoporous BCNs with tunable pores, surface areas, and nitrogen contents through a simple interaction of borane ammonia complex (BAC) with low‐cost sucrose molecules, combined with the hard‐templating approach. Nitrogen adsorption, powder X‐ray diffraction (XRD), and high resolution transmission electron microscopy (HRTEM) measures reveal a well‐ordered mesoporous structure with a high specific surface area. Mesoporous boron carbon nitride (MBCN) exhibits much higher specific capacitance as compared to bulk BCN and mesoporous carbon. It also displays a higher specific capacity for sodium‐ion battery and larger adsorption capacity for CO_2_ molecules as compared to the multiwalled carbon nanotube, mesoporous carbon, mesoporous silica, activated carbon, and mesoporous carbon nitrides. With the combination of excellent textural parameters, ordered porous structure, and tunable nitrogen contents, BCN offers the platform for various applications.

## 2 Results and Discussion

**Figure 1:**
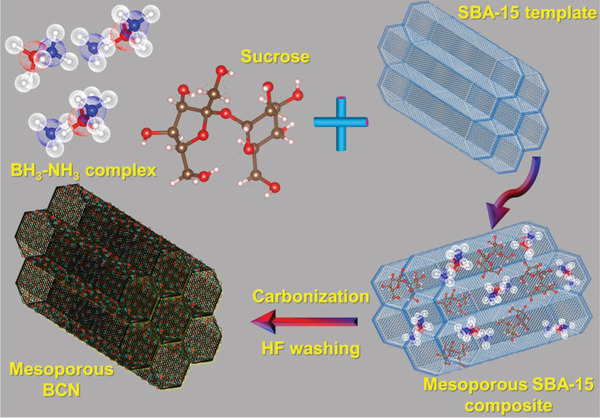
Schematic representation of the synthetic procedure for mesoporous BCN.

**Figure 2:**
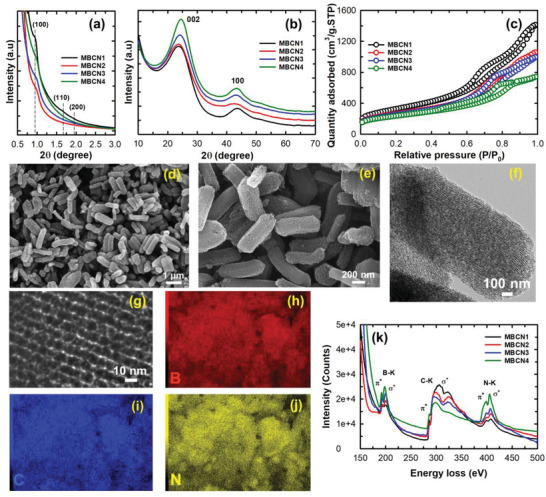
a) Low‐angle and b) high‐angle powder X‐ray diffraction patterns. c) N_2_ adsorption–desorption isotherm. d,e) SEM images showing rod‐like morphology of MBCN1. f,g) HRTEM images showing an ordered porous structure in MBCN1 and the corresponding EDX mapping of h) boron, i) carbon, j) nitrogen of MBCN1 sample, and k) EELS spectra of MBCN samples.

MBCNs were synthesized using Santa Barbara Amorphous‐15 (SBA‐15) (Supporting Information) as a template and sucrose, BAC as the carbon, boron, and nitrogen precursors. **Figure** **1** represents a schematic description of the synthesis process, and the detailed procedure is given in the Supporting Information. The samples are denoted as MBCN*x* where *x* denotes amount of BAC in gram. The low‐ and high‐angle XRD patterns of BCN synthesized with different BN concentrations are shown in **Figure** **2**. As depicted in Figure 2a, all these materials show a broad hump at the low angle that can be indexed to the (100), (110), (200) reflections of the 2D hexagonal space group *p*6*mm*, indicating that the well‐ordered mesoporous structure was replicated from SBA‐15. A similar mesoporous structure was previously observed for ordered mesoporous carbons (Figure S1a,b, Supporting Information). The ordered structure formation is attributed to the stable self‐assembled molecular structure formed within the nanochannels of the template through a simple hydrogen bonding between the BAC molecules and the hydroxyl groups of the sucrose molecules. Upon the heat treatment, the water molecules are removed to form a stable BCN network within the nanochannels of the template. The mesoporous structure of the template is replicated into the BCN network after removing the template with HF. It is noted that the low angle peak is shifted toward a higher angle as the amount of BAC is increased. When the amount of BAC is low, the *d*‐spacing of the sample is increased due to the incomplete filling of the pores with the BCN complex that expands the unit cell size upon the heat treatment and the template removal. Likewise, when the amount of BAC is reduced to 0.05 g, structural order collapses in BCN due to incomplete polymerization between BAC and sucrose molecules (Figure S2, Supporting Information). Wide‐angle XRD patterns of the samples display two broad peaks around 25° and 43° (CuK*α*), corresponding to the (002) and (100) lattice planes of BCN with a layered hexagonal boron nitride (h‐BN) like structure (Figure 2b).^[^ ^16^ ^]^ While increasing the amounts of BN precursor from 0.1 to 0.4 g, a broadening of the peak accompanied by a slight peak shift to the lower angle is observed,^[^ ^2^ ^]^ confirming the doping of BN in the carbon lattice that forms ternary B—C—N compound.

Nitrogen adsorption–desorption studies were performed to understand the mesoscale ordering and textural parameters of MBCN. MBCN samples display a type‐IV isotherm and exhibit H1 hysteresis with featured capillary condensation in the higher relative pressure region, indicating the presence of well‐ordered mesopores in all the samples (Figure 2c). For MBCN1, there are two distinct capillary condensation steps in the isotherm, confirming two types of interconnected mesopores. These bimodal pores are highly beneficial for adsorption and energy storage applications (Figures S3 and S4b, Supporting Information). The MBCN samples also show high specific surface areas (1165.9–626.5 m^2^ g^−1^) and large pore volumes (2.18–0.44 cm^3^ g^−1^). MBCN1 registers the highest specific surface area and the specific pore volume. It is interesting to note that the specific pore volume of MBCN1 is 2.18 cm^3^ g^−1^ which is much higher than that of ordered mesoporous carbon (1.75 cm^3^ g^−1^) (Figure S4a and Table S1, Supporting Information). The specific surface area and the pore volume decrease with the concomitant increase of the pore diameter as the amount of BAC is increased in the synthesis mixture (Table S1, Supporting Information). High resolution scanning electron microscopy (HR‐SEM) images of MBCN show uniform rod‐shaped morphology with the length varying between 400 and 600 nm and the width of 80–200 nm and uniform mesopores (Figure 2d,e and Figure S5 (Supporting Information)), similar to the template materials, confirming the successful replication of the mesoporous SBA‐15 template. Representative HRTEM images of sample MBCN1 at different magnifications reveal the presence of a well‐ordered mesoporous channel (Figure 2f,g), and this well‐ordered mesoporous matrix is seen throughout all the samples, as shown in Figure S6 (Supporting Information). From the images, it is endorsed that the structural order is fully conserved during the replication process, and the template elimination process did not destroy the mesoporous nature of BCN samples.

The nature and coordination of B, C, and N and their distributions were confirmed by high‐resolution electron energy loss spectroscopy (EELS) spectra (Figure 2k) and the energy dispersive X‐ray analysis (EDX) and elemental mapping, respectively (Figure 2h–j and Figure S7 (Supporting Information)). The elemental quantification of the integrated EELS signals commensurates with B_0.132_C_0.725_N_0.142_, B_0.168_C_0.655_N_0.176_, B_0.192_C_0.598_N_0.208_, and B_0.219_C_0.548_N_0.232_ stoichiometry in MBCN1, MBCN2, MBCN3, and MBCN4 samples, respectively. EELS spectra of the samples reveal K‐shell excitation edges of B, N, and C (Figure 2k). All the samples display a clear splitting of π* and σ* peaks attributed to the sp^2^ hybridization, further confirming graphitic‐like structure in the MBCN samples. A clear increase in the intensity of the N K‐shell excitation peak with the concomitant decrease of the C K‐shell excitation peak is observed when the amount of BAC is increased from 0.1 to 0.4 g. These results clearly endorse that our facile method can control the composition of B and N in MBCN with the simple adjustment of the amount of BAC. It should also be noted that BCN phase cannot be formed when the amount of BAC is reduced below 0.1 g.

**Figure 3:**
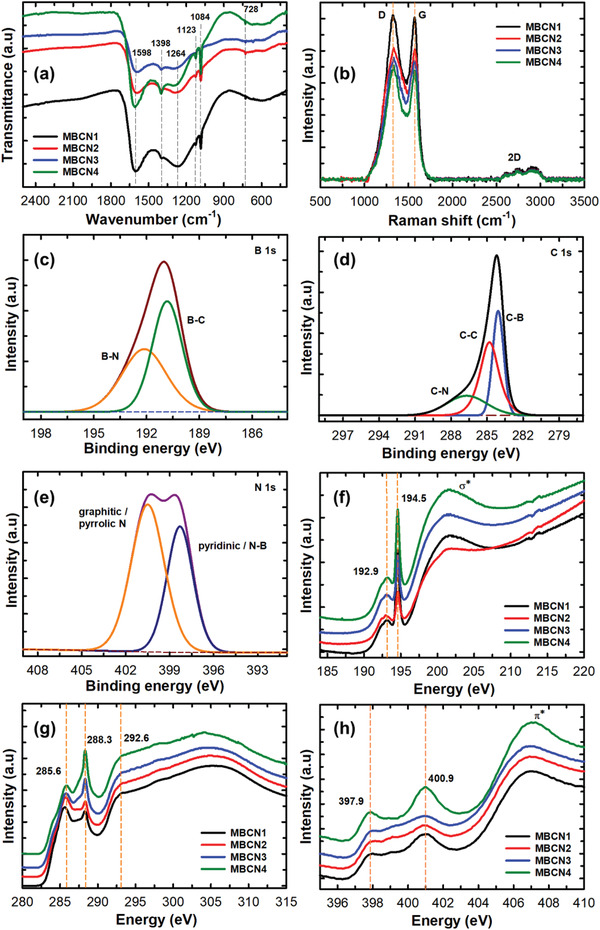
a) FTIR spectra of MBCN samples. b) Raman spectra of MBCN samples. c) High‐resolution XPS B 1s. d) High‐resolution XPS C 1s. e) High‐resolution XPS N 1s spectra. f) B K‐edge, g) C K‐edge, and h) N K‐edge XANES spectra.

Fourier transform infrared (FTIR) spectroscopy analysis was performed to obtain a deeper insight into the chemical structure of MBCN. The spectra of MBCN samples show B—N bond at 1398 and 728 cm^−1^ and B—C bond at 1264, 1123, and 1084 cm^−1^ for all the BCN samples (**Figure** **3**a). The broad and large absorption band after 728 cm^−1^ corresponds to the out‐of‐plane bending vibration of B—N—B bonds, whereas the small band at ≈1398 cm^−1^ attributes to the in‐plane transverse stretching vibration of the B—N bond, indicating that the hexagonal structure of h‐BN is maintained in the BCN structure.^[^ ^17^ ^]^ The peak at 1264 cm^−1^ is related to the B—C bond, which becomes smaller as the amount of the BN precursor is increased. The band at 1084 cm^−1^ corresponds to both B—C vibrations and sp^3^ stretching mode of B—N bands, confirming the nonexistence of the cubic phase of BN.^[^ ^18^ ^]^ The band located at ≈1598 cm^−1^ is assigned to the sp^2^ C—N bonds. Functional groups identified from the FTIR results clearly show the formation of BCN. The structure of MBCN was also analyzed using Raman spectroscopy. Figure 3b shows two obvious peaks at 1330 and 1570 cm^−1^, corresponding to the D‐band and G‐band of carbon, respectively. The G‐band arises from the bond stretching of sp^2^‐bonded pairs, while the D‐band is associated with the sp^3^ carbon of the defective site. As the amount of B and N is increased, a decrease in the intensity of D‐ and G‐band peaks is observed. The intensity ratio of *I*_D_/*I*_G_ also increases from 1.01 to 1.04, increasing the amount of B and N in the samples, confirming a higher ratio of carbon defects in MBCN4.^[^ ^19^ ^]^ Two small peaks at 2700 and 2900 cm^−1^ corresponding to the weak 2D‐ and D + G‐bands also suggest the existence of defects generated from the incorporation of B and N in the carbon framework.

X‐ray photoelectron spectroscopy (XPS) measurements were carried out to analyze the electronic structure and composition. The XPS survey spectra reveal the presence of boron, carbon, nitrogen, and oxygen present in the MBCN samples (Figure S8, Supporting Information). The small amount of oxygen that is increased with increasing the N content may be originated from the adsorbed oxygen on the surface. The core‐level spectrum of B 1s in Figure 3c (MBCN1) can be deconvoluted into two well‐resolved peaks that are centered at 190.9 and 192.3 eV. These peaks correspond to the B—C and B—N bonds, respectively.^[^ ^20^ ^]^ The B—C and B—N bond percentage calculated from the B 1s spectrum for MBCN1 is 54.3% and 45.7%, respectively. A similar percentage of B—C and B—N bonds is also observed for the samples MBCN2, MBCN3, and MBCN4 (Figure S9, Supporting Information). The high‐resolution XPS C 1s signal of the representative MBCN1 can be deconvoluted into three different peaks centered at 283.9, 284.7, and 287.2 eV, corresponding to B—C, sp^2^ carbons C—C, and C—N bonds, respectively (Figure 3d). From the C 1s spectrum deconvolution results, it is noticed that the C—C bond occupies 44.11%, while the B—C and C—N bonds possess 30.05% and 25.84%, respectively, demonstrating that the C—C bond amount is significantly higher than that of B—C and C—N bonds. While increasing the amount of BN precursor (Figure S10, Supporting Information), the C‐domain‐embedded matrix reduces, wherein the C—C bond decreases (44.11–37.2%) and the contribution of B—C and C—N bonds increases gradually to 30.89% and 31.91%, respectively (samples MBCN2–MBCN4). Thus, C 1s spectra support the effective incorporation of BN into the carbon, which is in accordance with the XRD results. N 1s spectrum of MBCN1 (Figure 3e) is deconvoluted into two contributions. The peak at 398.3 eV (39.6%) corresponds to the pyridinic nitrogen and N—B bonds,^[^ ^19^, ^21^ ^]^ while the peak at 400.3 eV (60.4%) attributes to pyrrolic N.^[^ ^19^ ^]^ It should be noted that the amount of pyrrolic N is decreased with the concomitant increase of N—B as the content of the N is increased in the MBCN samples (Table S2, Supporting Information). The peak at 398.3 eV is slowly merged with the N—B peak for MBCN3 and MBCN4. Elemental analysis shows that the composition of carbon and nitrogen varies (“C” – decreases, “N” – increases) by increasing the amount of B and N (Table S2, Supporting Information), suggesting a successful formation of BCN. The XPS deconvoluted data are in good agreement with the EELS analysis, which confirms the presence of ternary BCN.

The spectra of X‐ray absorption near‐edge spectroscopy (XANES) were measured for the MBCN samples to provide a broader view of the local bonding environment of each element. Figure 3f–h shows the B K‐edge, C K‐edge, and N K‐edge spectra of the MBCN samples. In the B K‐edge spectra, the sharp peak at 194.5 eV can be assigned to the B 1s → π* transition, showing a clear fingerprint of sp^2^ hybridization of the B—N bond formation. We also notice that the intensity of the peak increases as the amount of BAC increases in the samples from MBCN1 to MBCN4 and confirms the presence of pyridinic nitrogen that forms a strong sp^2^ bond with boron. A small broad peak at 192.9 eV showing π* transition indicates the dominant feature for higher nitrogen content. The broad peak in the range of 199.8–209 eV corresponds to the 1s → σ* transition, which specifies the splitting of the peak due to B—C and B—N bonds, and the same feature can be observed in all other samples.^[^ ^19^ ^]^ In the carbon K‐edge spectrum, two sharp peaks at 285.6 and 288.3 eV correspond to the 1s → π* transition, contributed from the graphitic, C—B bonds, and pyridinic nitrogen as in the CN framework.^[^ ^19^ ^]^ The broad peak at 292.6 eV matches the σ* transition composed of the C—C, C—B, and pyridinic C—N bonds. The bonds C—B and pyridinic C—N contribution seem to increase with the increase in the concentration of B and N in the samples, and the same feature has been observed from the XPS data of C 1s spectra. The broad peak above 300 eV appears due to σ* states that are interpreted to be due to the disorder and defect in the density of states (DOSs) (MBCN1 and MBCN2). The two broad peaks at 397.7 and 400.9 eV in nitrogen K‐edge arise from the two unfilled π* orbitals of pyridine and the broad peak centered at 407 eV is also assigned to the π* transition similar to pyridine and triazine.^[^ ^19^, ^22^ ^]^

**Figure 4:**
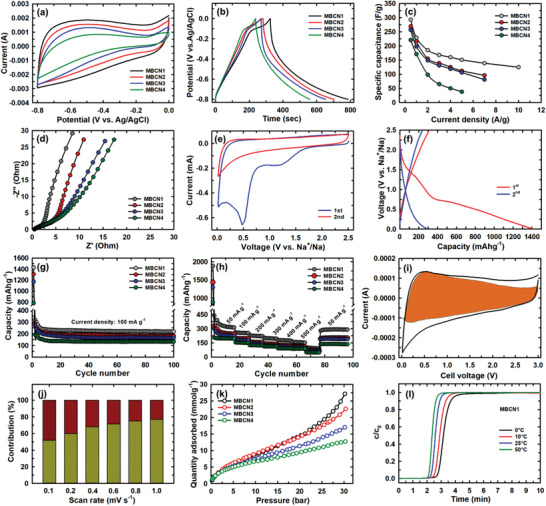
a) Cyclic voltammetry curves and b) charge–discharge profile of MBCN samples measured at 0.5 A g^−1^. c) Specific capacitance of MBCN sample at different current densities. d) EIS spectra. e) CV profiles of MBCN1 at a scan rate of 0.2 mV s^−1^. f) First discharge and second charge and discharge profiles of MBCN at a current density of 100 mAh g^−1^. g) Cycling performances with a Coulomb efficiency of MBCN1 at a current density of 100 mA g^−1^. h) Rate capability. i) Contribution of (pseudo)capacitive behavior (colored region) to the overall sodium storage. j) Normalized contribution of (pseudo)capacitive and diffusion‐controlled charge storage in MBCN1, yellow and brown colors represent (pseudo)capacitive and diffusion‐controlled contribution, respectively. k) CO_2_ adsorption isotherm of MBCN samples measured at 0 °C and l) breakthrough curves of MBCN1 samples at 0, 10, 25, and 50 °C.

MBCN samples were used to make electrodes for supercapacitors and sodium‐ion batteries to understand the energy storage performance. The supercapacitive properties were studied in a three‐electrode configuration and alkaline electrolyte (6 m KOH) media. **Figure** **4**a shows a distinctive cyclic voltammogram of MBCN samples measured at a scan rate of 20 mV s^−1^. Cyclic voltammograms measured at different scan rates are shown in Figure S10 (Supporting Information). The cyclic voltammetry (CV) curve of MBCN1 is quasirectangular in shape, while increasing the concentration of BN results in a pseudocapacitive behavior which is evident from Figure 4a of MBCN2–MBCN4 samples. These results show that the addition of N and B into the porous carbon matrix changes the electronic properties and favors the surface reaction and charge transfer, inducing the pseudocapacitive behavior in the MBCN samples. Galvanostatic charge–discharge curves were measured from 0 to −0.8 V at 0.5 A g^−1^, as shown in Figure 4b. The charge–discharge curve of MBCN1 shows a triangular shape resembling that of an ideal capacitor, whereas MBCN2–MBCN4 show a battery‐like pseudocapacitive behavior due to the increased C—N and B—N bonds in the carbon wall structure. Additionally, a nonlinear galvanostatic charge/discharge curve at around −0.1 V can be observed during charging that could be attributed to the redox reaction, which is in good agreement with the oxidation peak observed in the CV curve.^[^ ^23^ ^]^ At 0.5 A g^−1^, MBCN1–MBCN4 display a specific capacitance of 296, 269, 257, and 221 F g^−1^, respectively. In the case of MBCN1, high surface area with bimodal mesopores could increase the electrolyte/electrode contact area and provide more active sites beneficial for enhancing the diffusion of the electrolyte ions that lead to a high specific capacitance.^[^ ^24^ ^]^ Notably, the specific capacitance of the samples is ≈2.5 times higher than mesoporous carbon (121 F g^−1^ at 0.5 A g^−1^). Figure 4c shows the current density versus specific capacitance plot, where the specific capacitance decreases with an increase in the current density (Figure S11, Supporting Information). The specific capacitance decreases at a higher charge–discharge rate, which is due to the occurrence of depletion or saturation of the protons in the electrolyte, and only the outer surface can be utilized for the charge storage, resulting in a significant reduction in the specific capacitance.^[^ ^25^ ^]^ The phenomenon is very common in supercapacitors due to the sluggish kinetics of electrochemical activities under a high charge/discharge rate. The electrochemical impedance spectroscopy (EIS) data of MBCN1 show the lowest charge transfer resistance when compared to other MBCN samples (Figure 4d). The electrochemical series resistance of the MBCN samples is 0.50, 0.55, 0.88, and 0.99 Ω for MBCN1–MBCN4, respectively, indicating that the Ohmic resistance increases when the concentration of B and N increases in the MBCN samples.

As the prepared MBCN materials exhibit a unique bonding structure, ordered mesopores, and textural parameters, we applied them as anode materials for sodium‐ion batteries. Figure 4e shows the cyclic voltammogram of MBCN1 measured at 0.2 mV s^−1^ in the potential range of 0–2.5 V. The 1st cycle of the CV curve displays a distinct large reduction in the intensity of peaks around 1.2 and 0.56 V corresponding to the irreversible reaction between the sodium ion and the surface functional groups (B—N domains), the decomposition of the electrolyte, and formation of the solid electrolyte interface (SEI) layer. Also, the peak at 1.2 and 0.01 V is attributed to the sodium extraction from the nanopores and the sodium insertion or extraction at the interlayer of the MBCN material. Similar trends are also observed in MBCN2–MBCN4 samples, as shown in Figure S12 (Supporting Information). A long cathodic and anodic slope from 0.8 to 0.01 V and at 0.2 V indicates the excellent reversibility of Na^+^ insertion/extraction reaction in the rest of the cycles.^[^ ^26^ ^]^ The constant current charge–discharge measurements of the MBCN1 sample were measured at 100 mA g^−1^ between a potential window of 0–2.5 V (vs Na^+^/Na), as shown in Figure 4f. The charge–discharge profiles of repeated cycles almost overlapped, indicating that the structure of MBCN material is highly stable. The Coulombic efficiency of MBCN samples is very low in the first cycle of around 24.9%, whereas the second cycle almost reaches 100% (Figure S13, Supporting Information). This performance might be due to the irreversible sodium loss during the formation of the SEI layer at the beginning and a common phenomenon in carbon materials with a high surface area resulting from porosity, defects, and functional groups, which results in the initial capacity loss.^[^ ^27^ ^]^ The irreversible capacity loss in the initial cycles can be diminished by optimizing the binders or adding additives and surface treatment to form a stable solid electrolyte interface film before cycling.^[^ ^28^ ^]^ Figure 4g displays the cyclic stability of the MBCN samples measured at 100 mA g^−1^. MBCN1 delivered a reversible specific capacity of 237 mAh g^−1^ after 100 cycles, while MBCN2–MBCN4 exhibited 191, 175, and 142 mAh g^−1^, respectively. The rate capability of MBCN was measured at different current densities (Figure 4h). After numerous sodium insertion and extraction processes, a high reversible capacity was achieved for the MBCN1 sample. MBCN1 revealed excellent cyclic stability and delivered an average specific capacity of 349 mAh g^−1^ at 50 mA g^−1^, which is much higher than other heteroatom‐doped carbon samples reported (Table S3, Supporting Information). The boron‐doped 3D hierarchical porous carbon exhibited a capacity of 200 mAh g^−1^ at 100 mA g^−1^, B‐doped porous carbon with a termite net structure delivers a reversible charge capacity of 177 mAh g^−1^ at 100 mA g^−1^, and a charge capacity of 310 mAh g^−1^ at a specific current of 50 mA g^−1^ was reported for the boron‐doped graphene quantum dot material.^[^ ^29^ ^]^ Likewise, nitrogen‐doped biomass carbon and carbon nanofiber revealed a capacity of 152 mAh g^−1^ at 50 mA g^−1^ and 282 mAh g^−1^ at 100 mA g^−1^, respectively.^[^ ^27^, ^30^ ^]^ While nitrogen‐ and oxygen‐doped porous carbon and boron‐doped Sb/SbO_2_@reduced graphene oxide (rGO) composite performed better than boron and phosphorous dual‐doped carbon microspheres with a capacity of 221.9 mAh g^−1^ at 50 mA g^−1^.^[^ ^31^ ^]^ The excellent storage performance of MBCN1 could be ascribed to the hierarchical tunable mesoporous nanoarchitectonics, defects, and the expansion of the interlayer distance created by the heteroatom doping. The higher specific capacity of MBCN1 compared to MBCN2–MBCN4 should be associated with the higher surface area, unique bonding structure, and better ionic charge kinetics. All the samples exhibit better performance than pure mesoporous carbon (MC), suggesting that B and N in the porous carbon matrix offer more active sites and relatively low resistance for fast ion transport. To investigate the contribution of (pseudo)capacitive behavior in electrochemical property of present MBCN1, CV analyses with six different scan rates were carried out (Figure S14a, Supporting Information). Based on the equation of *I* = *αν^b^*, where *I* is the measured current, *ν* is the sweep rate, and *α*‐ and *b*‐values are variable parameters,^[^ ^32^ ^]^ *b*‐values were estimated as 0.86 and 0.77 at the cathode and anode sweeps, respectively (Figure S14b, Supporting Information), underscoring the partial contribution of (pseudo)capacitive behavior on sodiation/desodiation process (Figure 4i). Furthermore, based on the equation *i*(*V*) = *k*_1_ *ν* + *k*_2_ *ν* ^1/2^, the contribution of (pseudo)capacitive behavior could be quantified.^[^ ^32^ ^]^ At the scan rate of 1.0 mV s^−1^, MBCN1 appears to possess 77% of (pseudo)capacitive contribution, which is more dominant rather than a diffusion‐controlled contribution, as shown in Figure 4j. This behavior can be ascribed to the high surface area and the presence of a large number of active sites in the interconnected meso‐ and microporous channels of the MBCN structure.

We have demonstrated that the obtained MBCN has a high specific surface area, highly porous and stable structure with tunable mesopores, and ultra‐micropores that intrigue us to explore their potential for CO_2_ capture. Figure 4k shows the CO_2_ adsorption curves of the MBCN materials investigated with a 0–30 bar pressure range at 0 °C. The CO_2_ adsorption capacities of MBCN1–MBCN4 samples at 30 bar pressure range 27.14–12.71 mmol g^−1^. From Figure 4k, we can observe a rapid increase of CO_2_ uptake at the lower pressure range followed by a gradual increase in the high‐pressure region without saturation. The sample MBCN2 with more micropores shows a higher amount of CO_2_ adsorption at 1 bar (2.56 mmol g^−1^) than that of other samples (MBCN1, 2.36 mmol g^−1^; MBCN3, 2.43 mmol g^−1^; and MBCN4, 2.46 mmol g^−1^). The CO_2_ adsorption capacities of these materials at the low temperature are consistent with the trend of the *t*‐plot micropore area from the N_2_ adsorption analysis (Table S1, Supporting Information). In the high‐pressure region, the CO_2_ uptake follows a different trend. The sample MBCN1, which has a larger surface area and higher pore volume, shows higher CO_2_ adsorption until 30 bar pressure. Activated porous carbon derived using halloysite as a template,^[^ ^33^ ^]^ N‐doped carbon,^[^ ^34^ ^]^ and biomass‐derived carbons^[^ ^35^ ^]^ showed similar behavior. This scenario clarifies that at the lower pressure range, the acidic CO_2_ molecules are physisorbed to the micropore channels presented inside the mesopores of BCNs. An increase in pressure fills the CO_2_ molecules to the mesopores, and additionally, the heteroatoms embedded in the hexagonal porous wall channels physically bind more molecules, leading to a higher adsorption amount. Additionally, the basic functionalities were measured and calculated using the temperature programmed desorption (TPD) of CO_2_. The unique desorption peak centered around 100 °C could be attributed to the fact that the MBCN1 sample registers the highest specific surface area, which helps to adsorb a large amount of CO_2_ in the porous channels. However, these CO_2_ molecules are desorbed at low temperature due to their poor interaction with the surface of MBCN1 resulting from the lower number of nitrogen atoms as compared to other samples. As can be noticed, the intensity of the peak at low temperature decreased significantly as the amount of the nitrogen atom increased in the MBCN samples.^[^ ^36^ ^]^ It has been found that MBCN1 registers the highest basicity among the MBCN samples prepared. MBCN1 exhibits high basicity of 0.813 mmol g^−1^ (Figure S15a and Table S4, Supporting Information) that triggered high adsorption of the acidic CO_2_ molecules. It is also demonstrated that MBCN samples are robust and do not collapse even at a high pressure of 30 bar. The saturation limit is not achieved yet, which implies that these materials may accommodate more CO_2_ molecules into the mesoporous channels at pressures higher than 30 bar.

As MBCN1 samples showed the highest adsorption capacity (27.14 mmol g^−1^), temperature‐dependent adsorption studies of this sample at 0, 10, 25 °C with a pressure from 0 to 30 bar were carried out (Figure S15b, Supporting Information). The capacity of CO_2_ adsorption at 30 and 1 bar decreased to 20.19 and 2.01 mmol g^−1^ at 10 °C compared to 27.14 and 2.36 mmol g^−1^ at 0 °C, and the value further decreased to 17.90 and 1.64 mmol g^−1^ while increasing the adsorption temperature to 25 °C, indicating that low temperature and an exothermic adsorption phenomenon favor higher amount of CO_2_ adsorption.^[^ ^33^ ^]^ While comparing the results of MBCN with the other adsorbents, including ordered MC, activated carbon, carbon nanotubes, mesoporous silica, mesoporous carbon nitride, and halloysite, it is noticed that MBCN1 registers the highest adsorption capacity. For example, the adsorption capacity of MBCN1 is 1.58 times higher than that of MC, which registers only 17.2 mmol g^−1^ at 0 °C (Figure S15b, Supporting Information). CO_2_ adsorption of MBCN1 was further compared with different carbon‐based materials reported previously (Figure S15c, Supporting Information). It was found that the CO_2_ adsorption of MBCN1 in our work is much higher than reported. To understand the mechanism of CO_2_ adsorption between the adsorbent and CO_2_, the Clausius–Clapeyron equation was applied to the isotherms measured at 0, 10, 25 °C of MBCN1 to calculate the isosteric heat of adsorption (*Q*_st_). As shown in Figure S15d (Supporting Information), the *Q*_st_ value calculated for CO_2_ is in the range of 21.70–6.51 kJ mol^−1^. The isosteric heat of adsorption value at the low pressure is 21.70 kJ mol^−1^, suggesting that physisorption is a dominant interaction between adsorbent and CO_2_. With the increasing pressure and CO_2_ adsorption capacity, the heat of adsorption decreases, which may be due to the heterogeneous nature of adsorption sites. Similarly, CO_2_ breakthrough measurements (Figure 4l) of MBCN1 were measured at atmospheric pressure by varying temperatures from 0, 10, 25, to 50 °C using a concentration of 1 vol% CO_2_ mixed with helium gas. The amount of CO_2_ adsorbed was calculated from the difference between the empty and sample curve. It is clear from the figure that the amount of CO_2_ captured by the adsorption sites on MBCN1 increases with a decrease in adsorption temperature. The saturation time for adsorption is quite long at 0 °C (≈300 s) and significantly decreases at 50 °C (≈190 s). This could be attributed to the sample's high surface area (1165.9 m^2^ g^−1^) and large pore volume (2.14 cm^3^ g^−1^). Early breakthrough time will result in a less CO_2_ uptake (0.385 mmol g^−1^), and a longer time will ensue a higher uptake (0.696 mmol g^−1^). These results demonstrate that the material would be a good adsorbent material at low concentrations due to the fast‐kinetic uptake of CO_2_. It is observable from the CO_2_ capture studies that exceptional textural features such as high surface area, porosity, and micropores in MBCN samples strongly influence the CO_2_ adsorption, and additionally, the presence of heteroatoms enhances the interactions between adsorbent and CO_2_ molecules.

**Figure 5:**
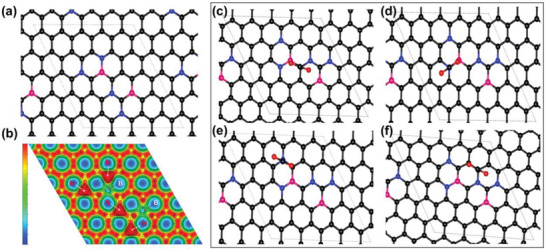
a) Optimized structure (blue, pink, and black represent nitrogen, boron, and carbon). b) 2D charge density. The CO_2_ adsorption profile of MBCN c) Model I, d) Model II, e) Model III, and f) Model IV (black, blue, and pink represent carbon, nitrogen, and boron, red represents O of CO_2_).

The CO_2_ adsorption ability of BCN has been proven to be higher than pristine graphene.^[^ ^37^ ^]^ It was previously demonstrated that the CO_2_ adsorption ability of BCN depends on the chemical bonding environment of the functional groups in the BCN framework.^[^ ^12^ ^]^ It was also highlighted that CO_2_ adsorption ability would be in the order of pyridone > pyridine > amine > quaternary > pyridine‐*N*‐oxide > cyanide > pyrrole functional groups. It was identified that in both pyridone and pyridine, CO_2_ preferred to adsorb at the adjacent carbon atom. However, the CO_2_ adsorption energy profile of graphene‐like B—C—N configuration is not yet studied. Hence in the present investigation, density functional theory calculations were carried out to investigate the CO_2_ adsorption property of MBCN samples. A 5 × 5 supercell of graphene aromatic ring structure was chosen to represent MBCNs. The number of C, B, and N atoms of MBCNs was chosen according to the C, B, and N concentrations identified from the XPS and EELS measurements. The optimized structure, band structure and DOS of MBCN1, MBCN2, MBCN3, and MBCN4 are shown in **Figure** **5**a and Figures S16 and S17 (Supporting Information). In the optimized structure of MBCN, the average bond length of B—C, B—N, C—C, and C—N is obtained as 1.48, 1.45, 1.42, and 1.38 Å, respectively. The relatively large B—C and B—N bond lengths lead to the increase in the lattice constant.^[^ ^12^, ^38^ ^]^ The simulated charge density profile of MBCN indicates that the electron is delocalized between the atoms. However, the electron density around B atoms is less compared to C and N, which might be due to the electron deficiency nature of B compared to C and N. Moreover, the charge density profile indicates that the N atom substituted on the adjacent B site behaves like pyridinic‐N and the N atom substituted on the adjacent C atom behaves like a graphitic‐N. The observed results indicate that position of N plays a critical role in the charge density profile.

Four different CO_2_ adsorption orientations were considered to probe the interaction between MBCNs and CO_2_ (Figure 5c–f). In Model I, Model II, Model III, and Model IV, the CO_2_ molecule is relaxed above the B—C, B—N, C—N, and C—C, respectively. The average distance between the adsorbed CO_2_ molecule and MBCNs is calculated as 3.2 Å. The calculated CO_2_ adsorption energy of MBCNs corresponding to Model I, Model II, Model III, and Model IV was −1.96, −1.94, −1.93, and −1.95 eV, respectively. The CO_2_ adsorption energy on MBCNs therefore follows the order of B—C > C—C > B—N > C—N. The negative adsorption values of all the models indicate that CO_2_ molecule can adsorb on all the sites of MBCNs and the most preferable site is on B—C. While, the lower CO_2_ adsorption energy of C—N compared to that of C—C supports the claim that CO_2_ molecule prefers to adsorb on the carbon adjacent to N. Therefore, the high adsorption of CO_2_ is due to the combination effect of high surface area and large active sites by B and N doping. As the higher doping concentration of B and N results in reduced surface area, it significantly weakens overall adsorption ability due to less number of adsorption sites. Therefore, MBCN1, which has the highest specific surface area and the reasonable amount of B and N, exhibits the highest adsorption ability for CO_2_ molecules.

## 3 Conclusion

In conclusion, we demonstrated a low‐cost synthesis approach for the preparation of well‐ordered MBCN with bimodal pores and tunable nitrogen contents with high specific surface area and large pore volume through an integrated approach of combining self‐assembly of low‐cost precursors and the nano‐hard‐templating approach. It was found that the specific surface area, pore volume, pore diameter, and nitrogen contents can be controlled through a simple adjustment of the amount of BAC in the synthesis mixture. The optimal MBCN sample exhibits a high specific surface area of 1166 m^2^ g^−1^ with a large meso‐ and micropore volume. We find that the optimized sample shows a specific capacitance of 296 F g^−1^ at 0.5 A g^−1^ in the aqueous medium, which is much higher than the carbon nanotubes (6 times) and ordered mesoporous carbons (2 times). We also demonstrate for the first time its potential as an anode for sodium‐ion battery with the specific capacity of 349 mAg h^−1^ at 50 mAh g^−1^ with nearly 100% Coulombic efficiency. In addition, MBCN shows an excellent CO_2_ adsorption capacity of 27.14 mmol g^−1^ at 30 bar pressure. We also demonstrated through density functional theory calculations that the combination of high textural parameters and the specific functional groups is required to achieve a high CO_2_ adsorption capacity. On the other hand, the high electrochemical performance of the MBCN samples is the direct contribution of the synergistic effect associated with the high surface area, enhanced ion transport kinetics, and B and N in the carbon active sites. We surmise that these nanostructured BCNs may provide new openings in the fabrication of innovative materials for both adsorption and energy storage and conversion applications.

## 4 Experimental Section

Experiment details are provided in the Supporting Information.

## Conflict of Interest

The authors declare no conflict of interest.

## Supporting information

Supporting Information

## Data Availability

The data that support the findings of this study are available in the Supporting Information of this article.
