# Supplementary material for: Ordered Mesoporous Boron Carbon Nitrides with Tunable Mesopore Nanoarchitectonics for Energy Storage and CO2 Adsorption Properties
Source: Adv Sci (Weinh). 2022 Apr 5;9(16):2105603. doi: 10.1002/advs.202105603 (PMC9165510; doi:10.1002/advs.202105603)
Supplement: Supplementary file 1 — Supporting Information [file ADVS-9-2105603-s001.pdf]

WILEY-VCH

ADVANCED SCIENCE
Open Access

Supporting Information

for *Adv. Sci.*, DOI: 10.1002/advs.202105603

Ordered Mesoporous Boron Carbon Nitrides with Tunable Mesopores Nanoarchitectonics for Energy Storage and CO_2 Adsorption Properties

*CI Sathish *, Gopalakrishnan Kothandam , Premkumar Selvarajan , Zhihao Lei, Jangmee Lee, Jiangtao Qu, Ala'a H. Al-Muhtaseb, Xiaojiang Yu, Mark B H Breese, Rongkun Zheng, Jiabao Yi*, Ajayan Vinu**

Supporting Information

Ordered Mesoporous Boron Carbon Nitrides with Tunable Mesopores Nanoarchitectonics for Energy Storage and CO_2 Adsorption Properties

*CI Sathish *, Gopalakrishnan Kothandam , Premkumar Selvarajan , Zhihao Lei, Jangmee Lee, Jiangtao Qu, Ala'a H. Al-Muhtaseb, Xiaojiang Yu, Mark B H Breese, Rongkun Zheng, Jiabao Yi*, Ajayan Vinu**

Dr. CI Sathish , Dr. Gopalakrishnan Kothandam , Dr. Premkumar Selvarajan, Mr. Zhihao Lei, Dr. Jangmee Lee, Prof. Jiabao Yi, Prof. Ajayan Vinu
Global Innovative Centre for Advanced Nanomaterials (GICAN), College of Engineering, Science and Environment, The University of Newcastle, Callaghan, NSW 2308, Australia.
* E-mail: ajayan.vinu@newcastle.edu.au; E-mail:sathish.ci@newcastle.edu.au;
E-mail: jiabao.yi@newcastle.edu.au

Dr. Jiangtao Qu, Prof. Rongkun Zheng
School of Physics, the University of Sydney, New South Wales 2006, Australia.

Prof. Ala'a H. Al-Muhtaseb
Department of Petroleum and Chemical Engineering, College of Engineering,
Sultan Qaboos University, Muscat, Oman.

Dr. Xiaojiang Yu, Prof. Mark B H Breese
Singapore Synchrotron Light Source, National University of Singapore, Singapore 117603, Singapore

Prof. Mark B H Breese
Department of Physics, National University of Singapore, 117542, Singapore.

Experimental

Synthesis of SBA-15 template

The mesoporous silica template SBA-15 was synthesized using a soft templating approach maintained at a strong acidic condition. In a typical synthesis, Pluronic P-123 (4 g) and a non-ionic surfactant, which is a tri-block copolymer ($EO_{20}PO_{70}EO_{20}$, mol.mass = 5750), were added to deionized water (30 g) and stirred continuously for 4 h at room temperature. 2M HCl (120 g) was then gradually added, and the temperature was increased to 40 °C with continued stirring for another 2 hrs. Subsequently, TEOS (9 g) was added slowly and retained in a static condition for 24 h. The mixture was transferred to a Teflon-lined autoclave and kept for hydrothermal treatment in an oven maintained at 150 °C for 48 h. The resulting precipitated material was filtered, washed several times with water, dried for 6 h at 100 °C, and finally calcined to remove the polymeric surfactant at 540 °C.

Synthesis of Mesoporous BCN

Mesoporous BCN was prepared in a hard templating method using sucrose and boron ammonia complex (BAC) as carbon, boron, and nitrogen source. In a typical synthesis, 1g of SBA-15 replica was pore filled using 0.5 g of sucrose and BAC (0.1 to 0.4 g) dissolved in 4 g water containing 0.1 ml sulphuric acid. It should be noted that we also tried BAC 0.05 g. However, characterization indicated that BCN could not be formed. The thoroughly mixed gel was carbonized at 900 °C for 3 h under a nitrogen atmosphere. The carbonized samples were then treated with HF solution (5 wt %), and the samples were washed several times with excess ethanol and dried at 100 °C for overnight. The as-prepared samples were labeled as MBCNx, where x denotes the percentage of BAC. For a comparison, mesoporous carbon (MC) was also synthesized by pore-filling 1 g of SBA-15 using a solution containing 0.5 g sucrose in 4 g water and 0.1 ml H_2SO_4.

Characterization

Powder X-ray diffraction (XRD) instrument Empyrean from Panalytical was employed for the phase characterization. The X-rays irradiated with CuKα radiation (λ = 1.5406 Å and 1.5444 Å) operating at a voltage and current of 40 kV and 40 mA, respectively. The low and high angle XRD patterns were obtained within a range of 0.7-5° and 10−70° 2θ using a step size of 0.007 and a scan speed of 150 seconds. Micromeritics ASAP 2420 adsorption instrument was used to investigate the N_2 adsorption at the liquid nitrogen temperature (80

K). All the samples were degassed at 200 °C for 12 hr, and the total surface area was calculated using the Brunauer-Emmett-Teller (BET) method. The surface morphology of the prepared materials was investigated using a scanning electron microscope (SEM,JSM-7900 F, JEOL) operating at a voltage of 2 kV and the transmission electron microscope (TEM, JEM-2100, JEOL) imaging was carried out at a voltage of 200 kV for the microstructure analysis. Fourier Transform Infrared (FTIR) analysis was performed (PerkinElmer instrument) by mixing a small amount of potassium bromide and MBCN samples with a ratio of 200:1. The mixture were pressurized to obtain a translucent disc, which was scanned in the range of 400–2500 cm^{-1} with a resolution of 1 cm^{-1} to obtain the spectra. Raman spectroscopy (9 XploRA, Horiba) was used to probe the surface bonding information of the samples using continuous 532 nm laser excitation. The Raman spectra were collected from 500-3500 cm^{-1} using a TE cooled CCD camera (Syncerity) attached to the monochromator of a spectrometer with 600 gr/mm. The spectra were obtained by collecting 120 s acquisitions. X-ray photoelectron spectroscopy (XPS, Thermo Scientific) was used for the analysis of valence state and composition of the samples using an Al Kα X-ray source (1486.7 eV) radiation source under an ultra-high vacuum (10^{-8} Pa) at room temperature. The core-level and high-resolution XPS spectra of the target elements B 1s, C 1s, N 1s, and O 1s were obtained and deconvoluted using the CasaXPS software. A PerkinElmer instrument was employed for the bulk elemental (CHN) composition analysis. X-ray absorption near-edge structure (XANES) analyses were carried out in Singapore Synchrotron Light Source (SSLS). Using a BELCAT II catalyst analyzer from MicrotracBEL, TPD of CO_2 was performed. Before measurements, the samples were outgassed at 200 °C in a helium atmosphere for 3h, cooled to 25 °C, and saturated with 1% CO_2 in helium for an hour. The physically adsorbed CO_2 was removed with helium isothermally. A TCD was used to monitor the amount of CO_2 desorbed while heating to 500 °C at a ramping rate of 5 °C min^{-1}.

Supercapacitor measurements

The supercapacitor measurements were carried out in a CHI760E (CH instruments) work station. To fabricate working electrodes, 10 mg of the sample was dispersed in 1 mL of ethanol, and subsequently, 50 μL of 0.5 wt% Nafion solution was added as binder. The prepared slurry was drop cast onto a nickel mesh and pressed in a roller after solvent evaporation. The prepared electrodes' supercapacitor performance was studied using aqueous

6 M KOH as an electrolyte solution in a three-electrode configuration. Pt wire and Ag/AgCl were used as counter and reference electrodes

Battery measurements

The battery measurements were done in a half-cell configuration. The anode electrode was fabricated for the electrochemical study by mixing 80 wt.% active material, 10 wt.% acetylene black, and 10 wt.% mixture with polyacrylic acid and carboxymethyl cellulose as a binder. The electrode slurry was then coated on a copper sheet with an electrode thickness of approximately 18 µm and dried at 120 °C for overnight. The battery cell was assembled using the 2032 standard coin cell in an Ar-filled glove box. A mixture of ethylene carbonate and dimethyl carbonate containing 1 M $NaClO_4$ was used as the electrolyte, and a thin ceramic polypropylene membrane was used as the separator. Sodium metal (Sigma Aldrich) was used as a counter and reference electrode. Galvanostatic charge/discharge measurements were carried out using WBCS3000Le32 (Seoul, South Korea), and all other electrochemical measurements, including cyclic voltammetry and EIS, were performed using Zive MP2 (Seoul, South Korea).

CO_2 adsorption measurements

The CO_2 adsorption was investigated using the HPVA (high-pressure volumetric analyzer) instrument from Micromeritics. Samples were degassed under vacuum at 200 °C prior to analysis for overnight to remove any impurities. All the samples were analyzed at 0 °C, and the pressure range was 0–30 bar. As the MBCN1 sample showed better adsorption than other samples, further studies at temperatures of 10 and 25 °C were carried out, and the isosteric heat of adsorption was calculated using the Clausius-Clayperon equation. The same material was used repetitively for measuring at different temperatures after degassing at a temperature of 200 °C under vacuum. Additionally, the CO_2 adsorption capacity was measured dynamically for the MBCN1 sample using a Microtrac BEL instrument. Each sample was pre-treated at 200 °C in a helium atmosphere for approximately 5 h. Around 100-150 mg of the material was packed in a quartz flow cell closed to both sides using quartz wool to make an adsorbent bed. The CO_2 adsorption was measured at four different temperatures of 0, 10, 25, and 50 °C and atmospheric pressure using a mixture of 1 % CO_2 and 99 % He gas with a flow rate of 30 mL min^{-1}. The background adsorption was performed without the sample under similar conditions. The amount of CO_2 adsorbed was calculated from the area of the

graph obtained by the subtraction of the sample breakthrough plot and the empty cell breakthrough plot.

Computational methods

All the first-principles calculations are done by the Vienna ab initio simulation package (VASP).[1] The core electron of all the atoms is represented by a projector augmented wave (PAW) basis set.[2] Perdew, Burke, and Ernzerhof (PBE) exchange-correlation functional within generalized gradient approximation (GGA) with a kinetic energy cutoff of 620 eV was used in all the calculations.[3] The geometrical structure of MBCNs was optimized by relaxing atomic positions and lattice vectors without any symmetry restrictions. A conjugate gradient scheme is used for the optimizations. 5 x 5 x 1 and 9 x 9 x 1 Monkhorst–Pack k-points mesh was used for total energy and DOS calculation. Hexagonal high symmetry first Brillouin zone was considered for band structure calculation. A vacuum space of 20 Å was used in the Z direction to avoid interactions between neighboring slabs. In the CO_2 adsorption calculation, the van der Waals (VdW) correction proposed by Grimme (DFT-D2) was used to approximate the dispersion correction.[4] A vacuum space of 25 Å was used for all the CO_2 adsorption calculations.

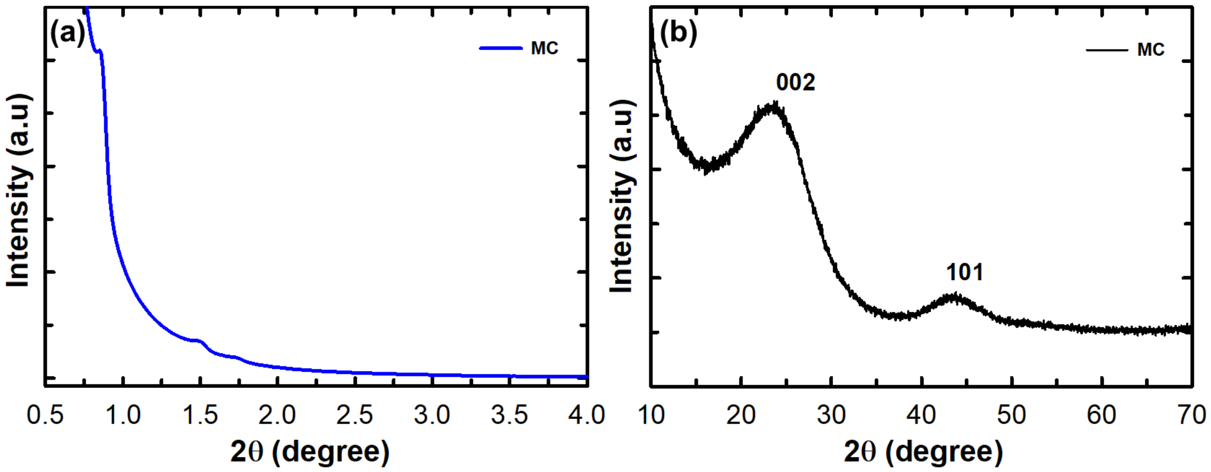

Figure S1. Powder X-ray diffraction of mesoporous carbon (MC): (a) Low angle and (b) High angle.

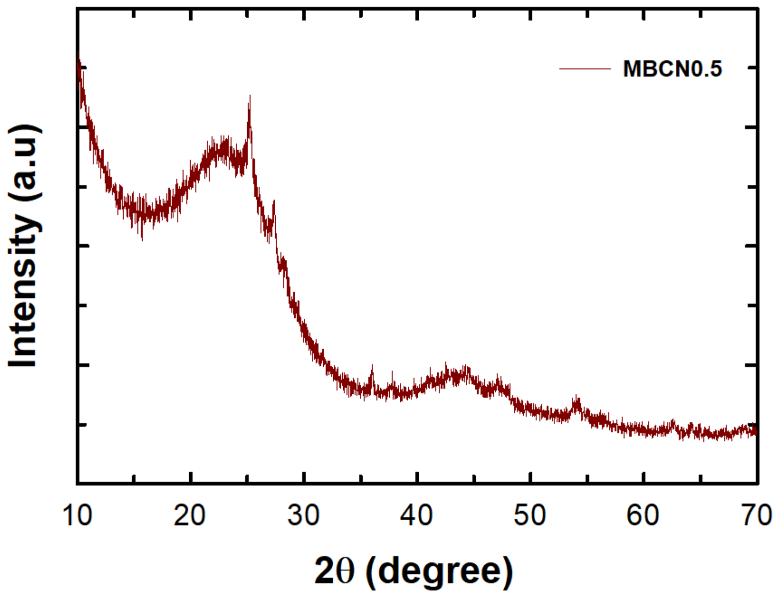

Figure S2. Powder X-ray diffraction of MBCN0.5.

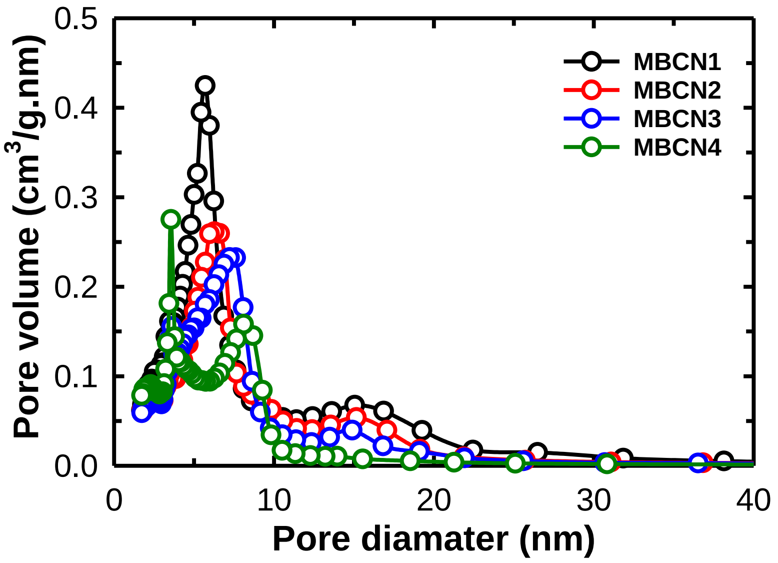

Figure S3. BJH adsorption pore size distribution.

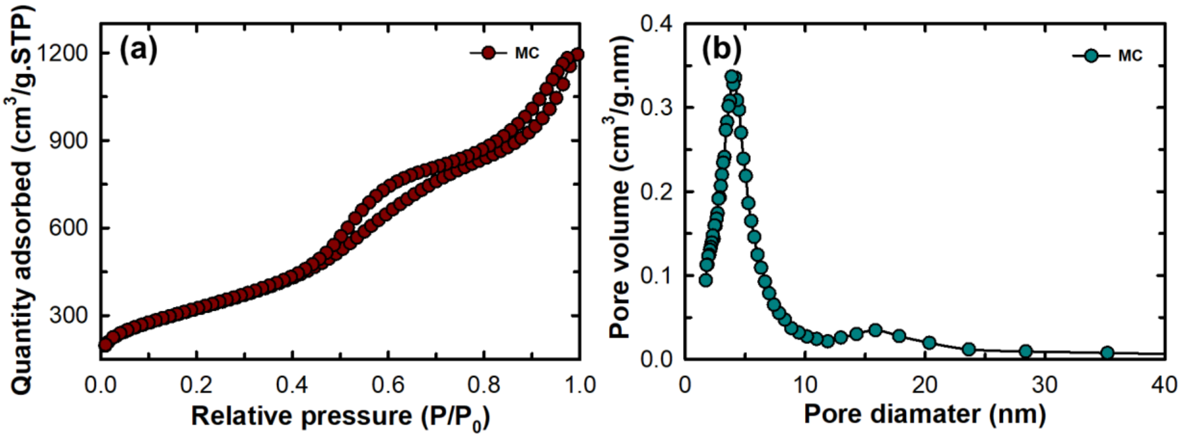

Figure S4. (a) N_2 adsorption-desorption isotherm. (b) BJH adsorption pore size distribution of mesoporous carbon.

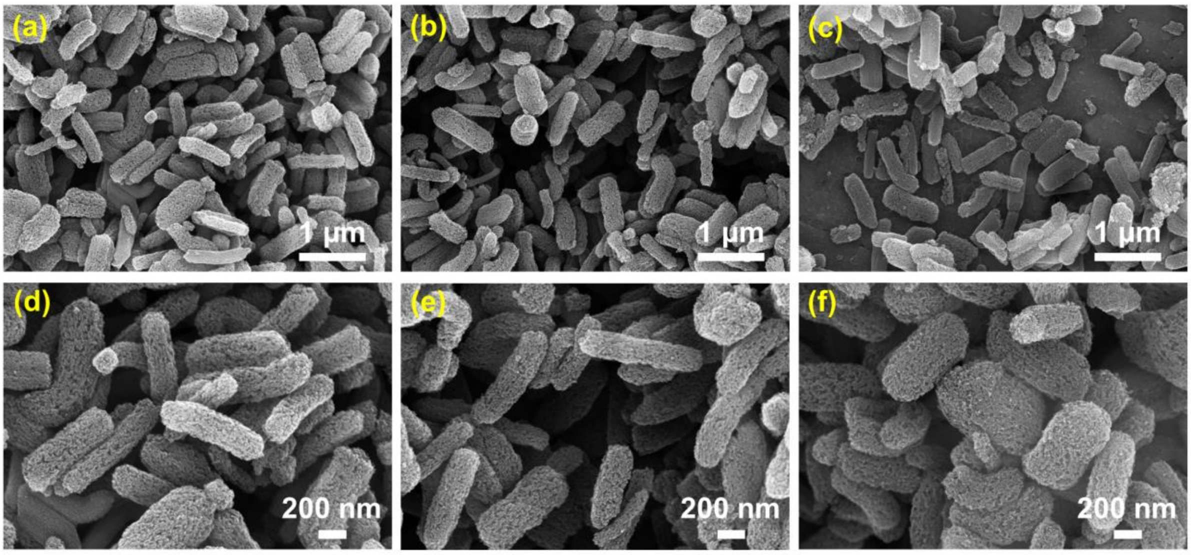

Figure S5. SEM images of (a, d) MBCN2, (b & e) MBCN3, and (c & f) MBCN4

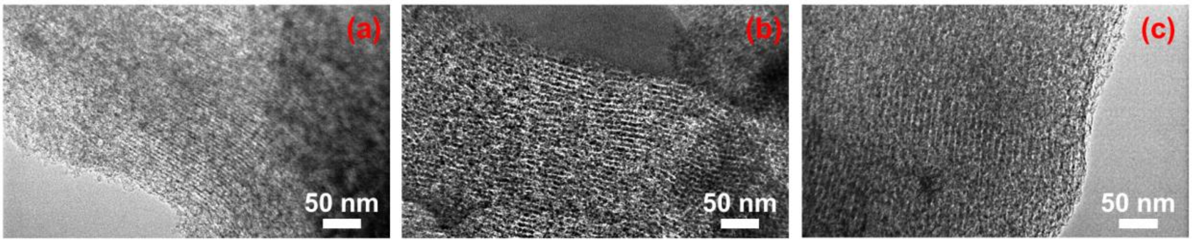

Figure S6. TEM images of (a) MBCN2, (b) MBCN3 & (c) MBCN4.

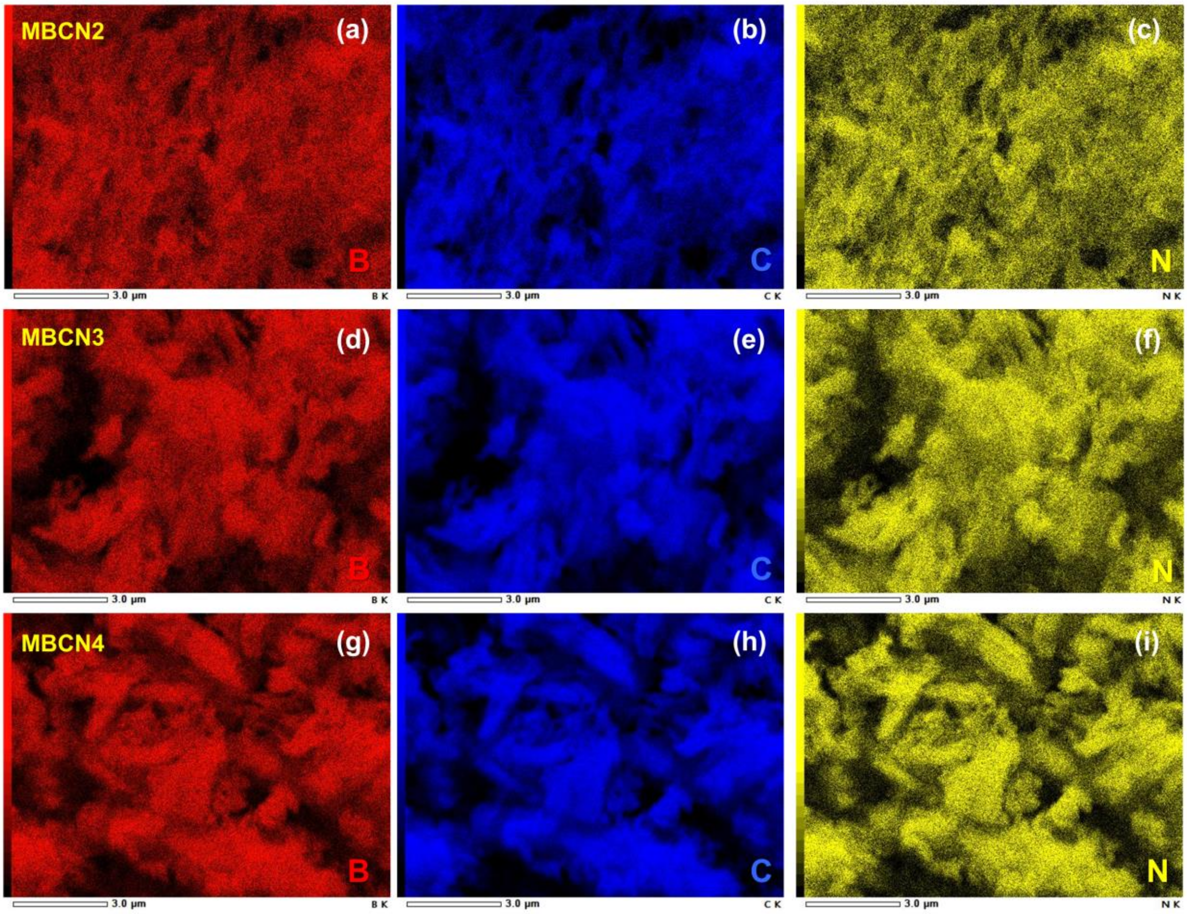

Figure S7: EDX mapping of (a, d & g) boron, (b, e & h) carbon, and (c, f & i) nitrogen of MBCN2, MBCN3, and MBCN4 samples.

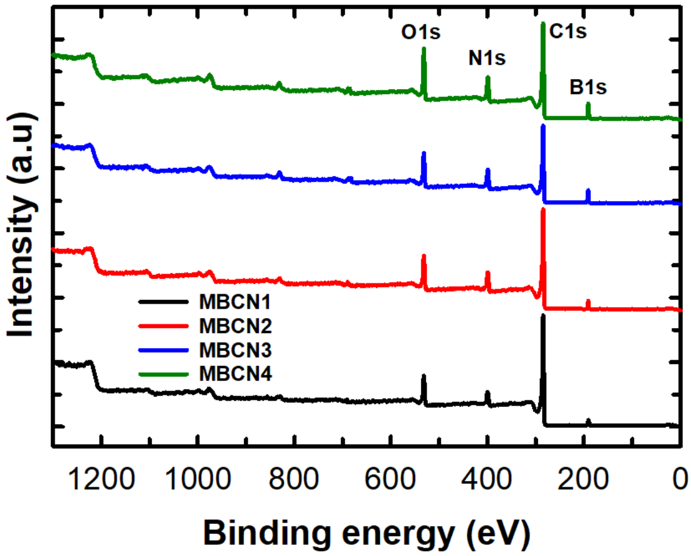

Figure S8. XPS survey spectrum of MBCN samples.

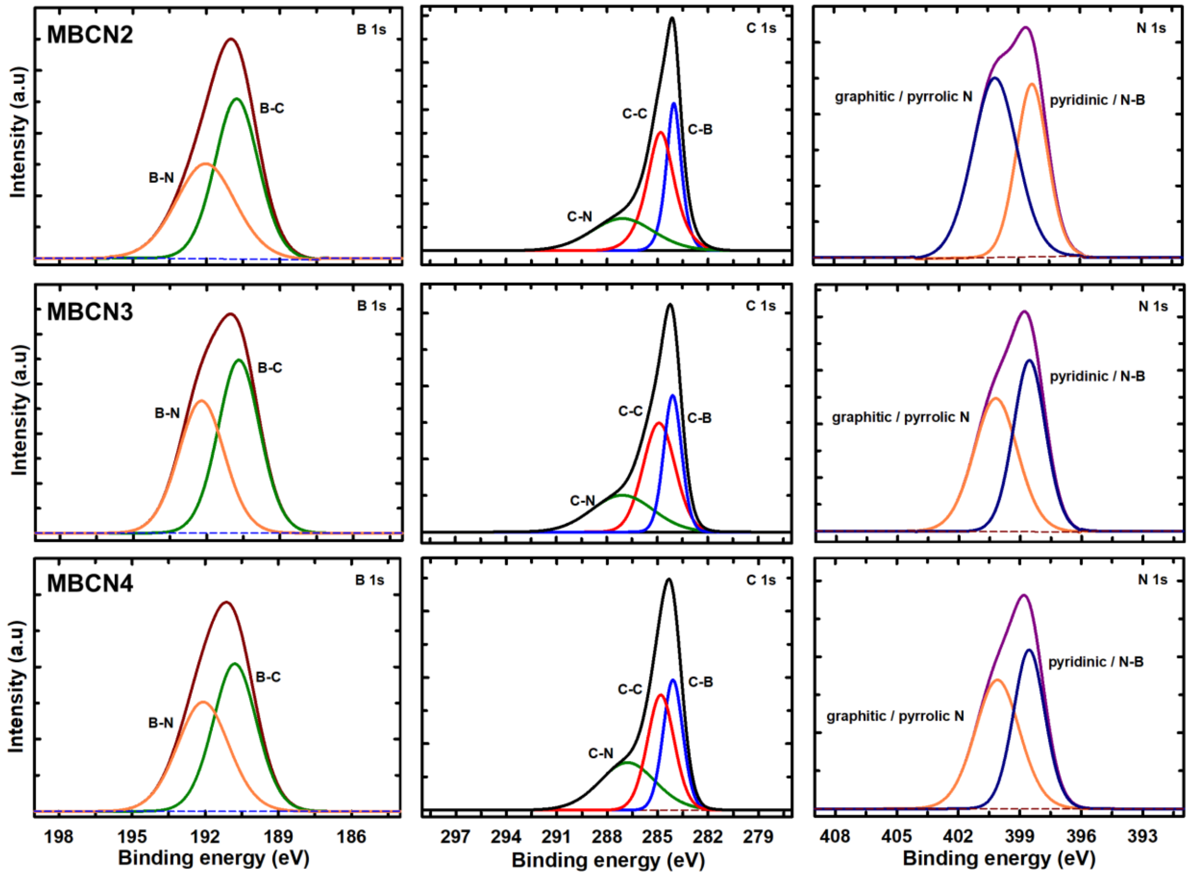

Figure S9. High-resolution XPS images of B 1s, C 1s, and N 1s spectra.

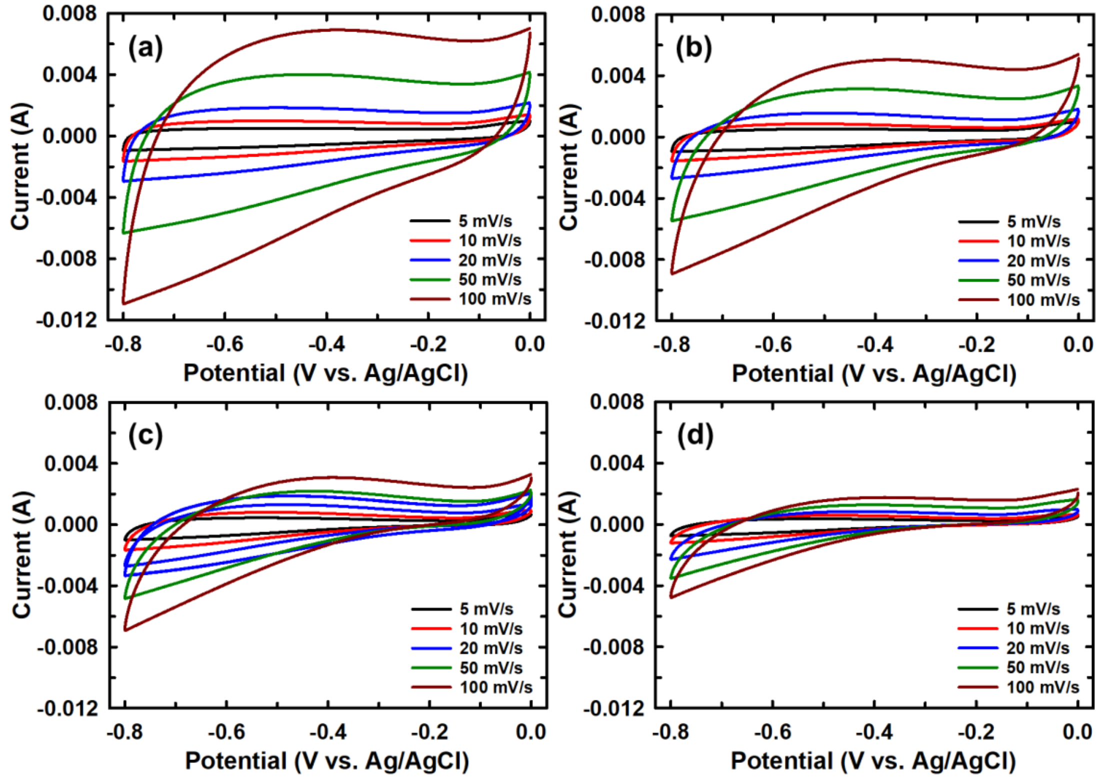

Figure S10. Cyclic voltammetry curves of (a) MBCN1, (b) MBCN2, (c) MBCN3, and (d) MBCN4 at different scan rates.

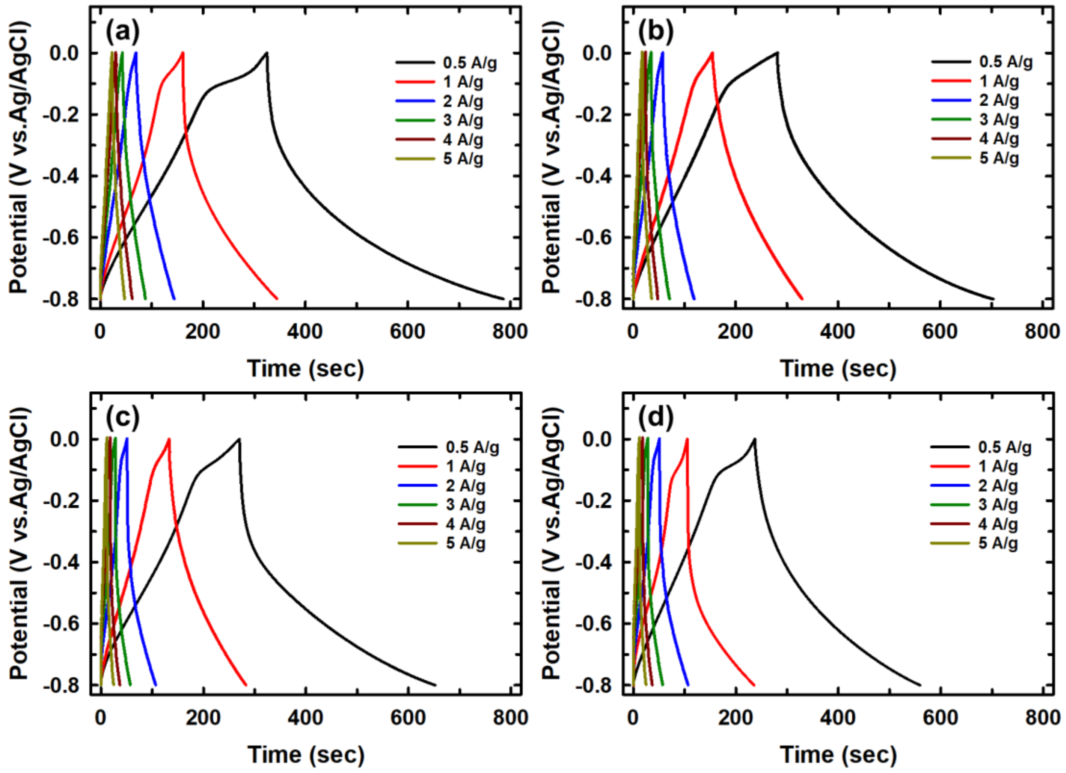

Figure S11. Charge-discharge profile of (a) MBCN1, (b) MBCN2, (c) MBCN3, and (d) MBCN4 at different current densities of 0.5-5 A/g.

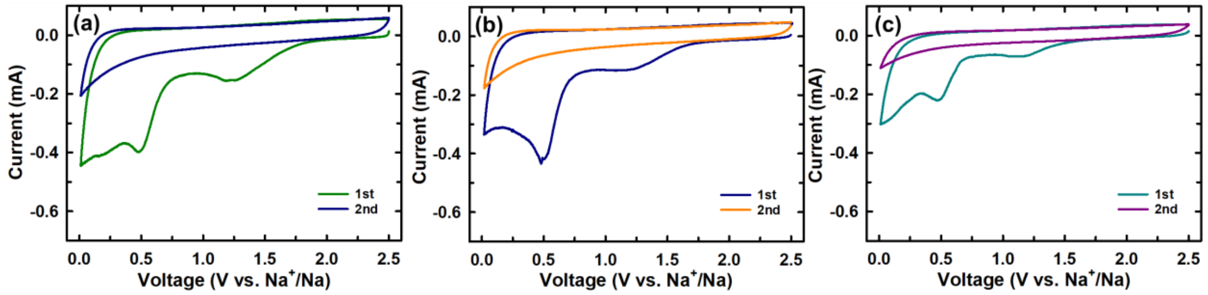

Figure S12. CV profiles of (a) MBCN2, (b) MBCN3, and (c) MBCN4 at a scan rate of 0.2 mVs^{-1}.

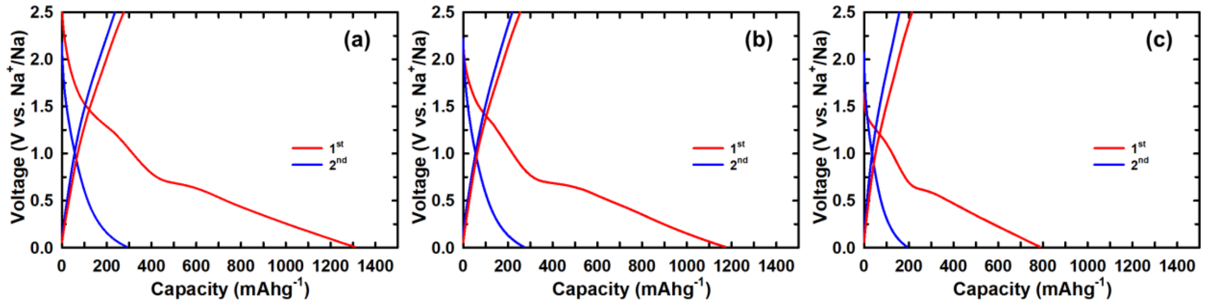

Figure S13. First discharge and second charge and discharge profiles of (a) MNCN2, (b) MBCN3 and (c) MBCN4 measured at 0.1 A g^{-1}.

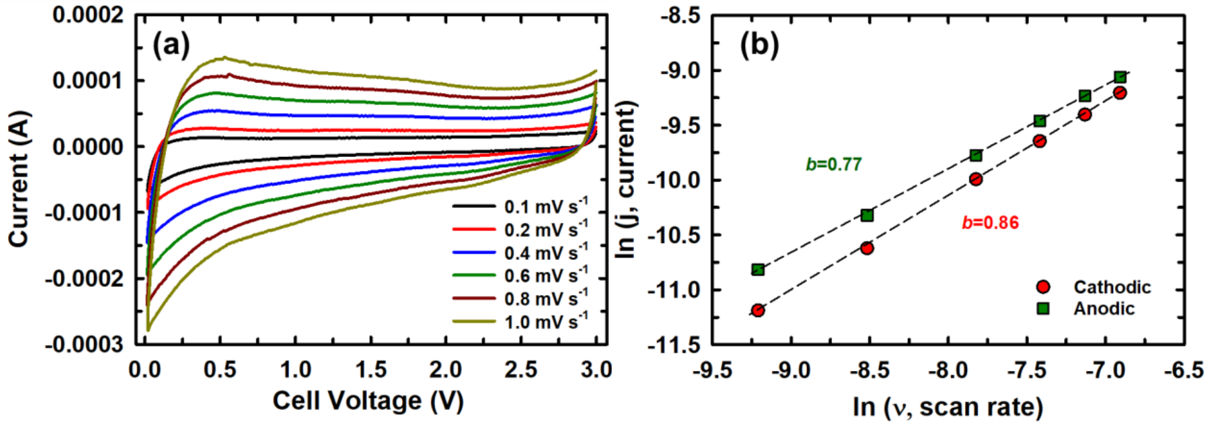

Figure S14. (a) CV profiles of MBCN1. (b) b-values by plotting the logarithmic peak current versus the logarithmic scan rate.

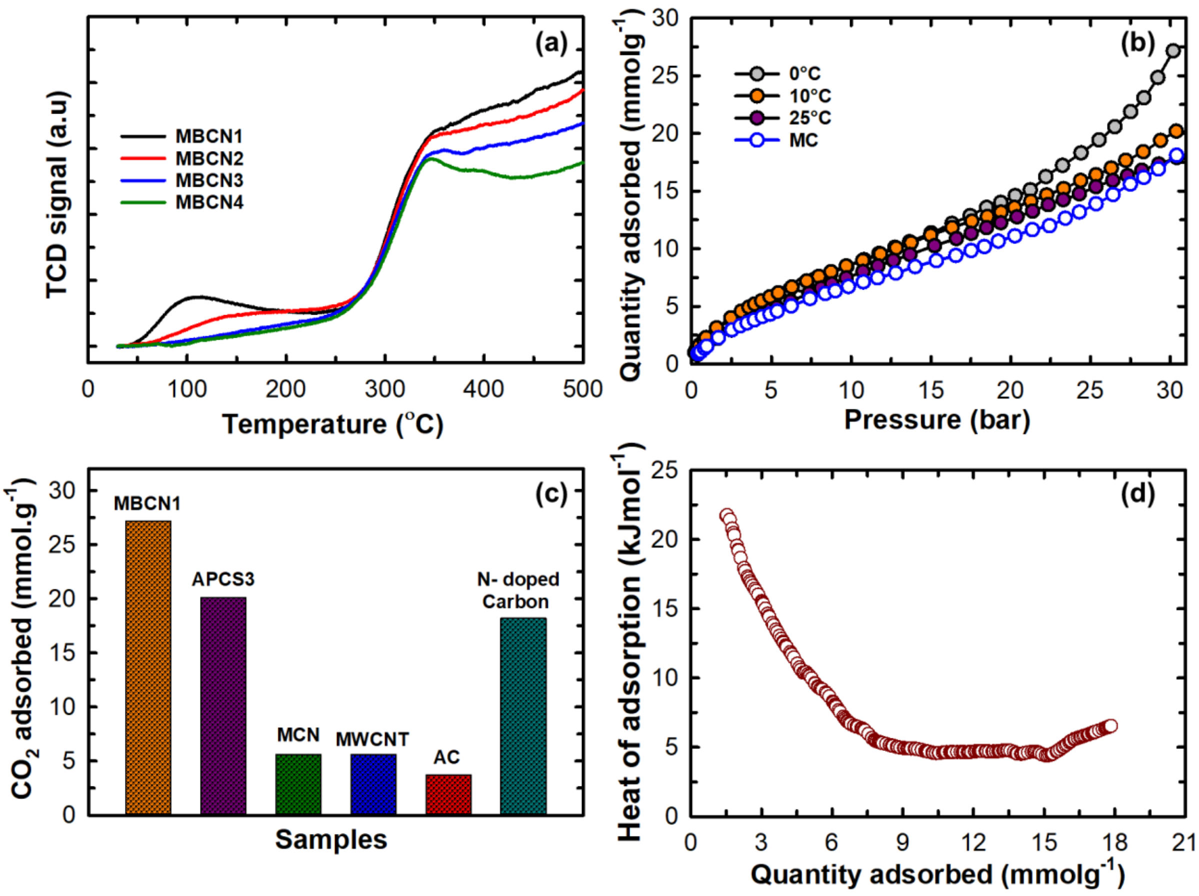

Figure S15. (a) TPD profiles of MBCN samples, (b) CO_2 adsorption isotherms of MBCN1 at different temperatures, and MC at 0 °C. (c) Comparison of CO_2 adsorption between MBCN1 and different carbon-based materials (MBCN1- mesoporous boron carbon nitride, APCS3- activated porous carbon, MCN-mesoporous carbon nitride, MWCNT- multi-walled carbon nanotube, AC-activated carbon[5]), and (d) Isosteric heat of adsorption of MBCN1 sample.

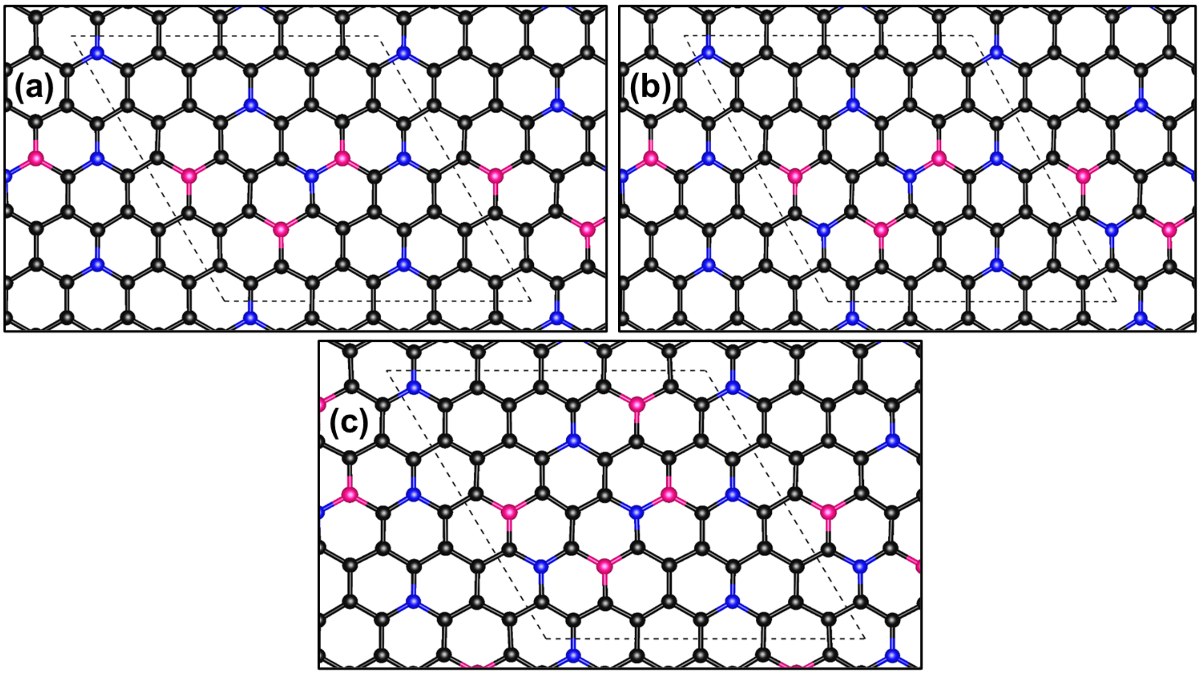

Figure S16. The optimized structure (black, blue, and pink represents carbon, nitrogen, and boron) of (a) BCN2, (b) BCN3, and (c) BCN4

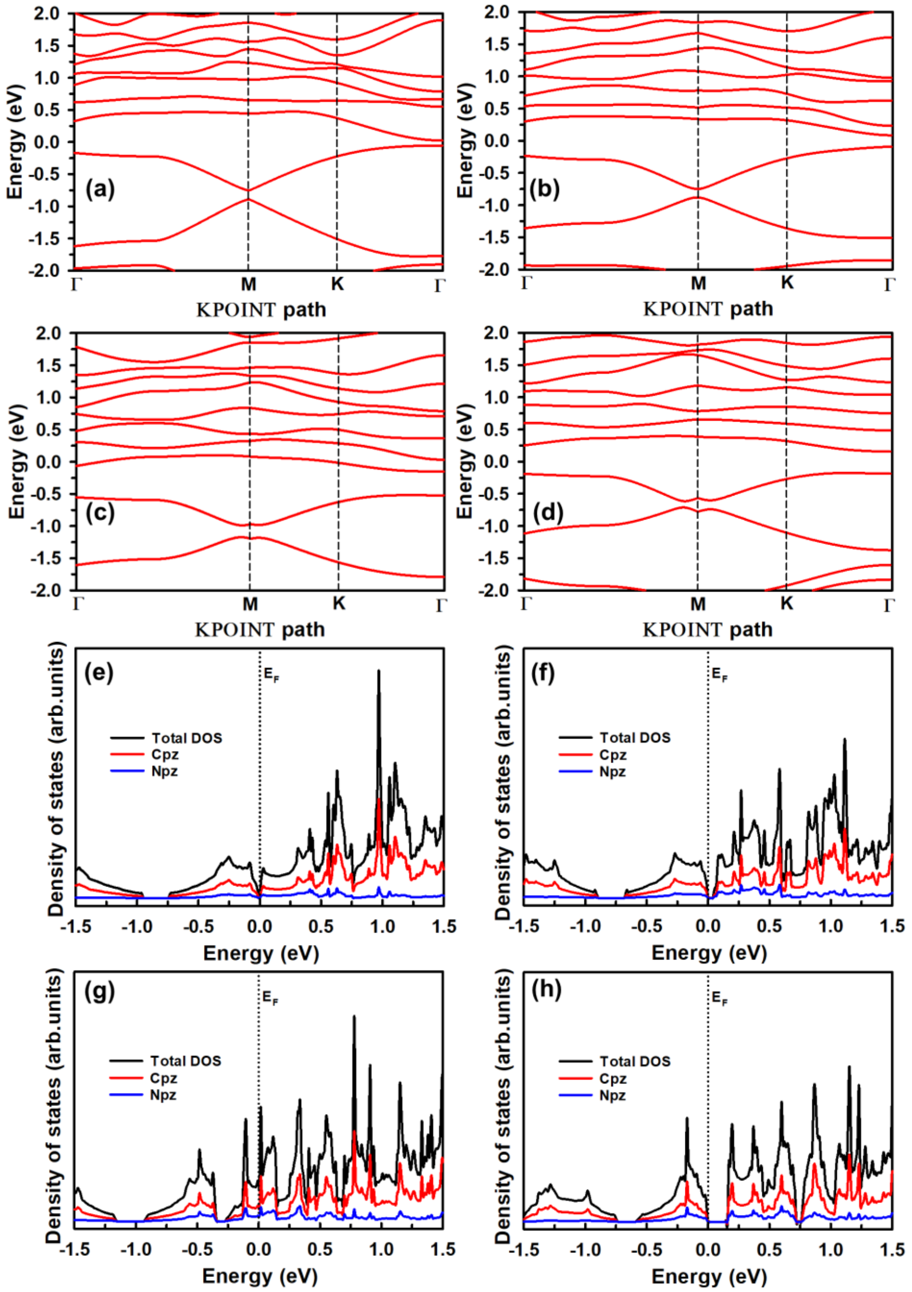

Figure S17. (a, b, c & d) Band structure and (e, f, g & h) DOS of MBCN1, MBCN2, MBCN3, and MBCN4 samples.

The calculated band structure and DOS of MBCNs are shown in Figure S17 (a-h), respectively. In general, B substitution leads to opening the bandgap below the Fermi level (p-type doping), whereas N substitution results in opening the bandgap above the Fermi level (n-type doping). The DOS of MBCNs indicates that the Fermi level is close to the conduction band (CB), which indicates that the MBCNs has n-type doping and higher N content is responsible for the n-type doping. The partial DOS reveals that in all MBCNs, the Cp_z and Np_z orbitals influence the valence band (VB) and conduction band (CB). The bandgap of MBCN1 is calculated to be 0.11 eV, which is slightly smaller than the experimentally observed value since DFT approximation usually underestimates the energy band gap**.**

Table S1: Textural parameters of MBCN samples from BET measurements

Sample Name	SA_{BET} (m^2g^{-1})	SA_{Micro} (m^2g^{-1})	PV (cm^3g^{-1})	PD (nm)		d-spacing (nm)
				Primary mesopore diameter	Large mesopore diameter	
MC	1261.68	121.41	1.75	4.2	15.68	0.346
MBCN1	1165.99	144.13	2.18	5.68	15.24	0.358
MBCN2	953.73	162.01	1.63	6.46	14.96	0.359
MBCN3	761.63	153.89	0.72	7.41	14.84	0.360
MBCN4	626.50	148.75	0.44	3.52	8.35	0.361

SA = Surface area; Micro = microporous; PD = Pore Diameter; PV = Pore Volume.

Table S2: Elemental analysis of MBCN samples showing composition of C, H & N

Sample	CHN data	XPS data						
		Boron		Carbon			Nitrogen	
		B-C	B-N	C-C	C-B	C-N	N-C/ N-B	graphitic/ pyrrolic N
MBCN1	69.55/1.02/14.9	54.3	45.7	44.11	30.05	25.84	39.6	60.4
MBCN2	64.43/1.09/17.37	55.2	44.8	43.47	31.63	24.90	40.5	59.5
MBCN3	53.35/1.63/21.02	54.1	45.9	42.65	29.97	27.38	43.6	56.4
MBCN4	49.11/1.99/23.76	53.4	46.6	37.2	30.89	31.91	46.6	53.4

Table S3: Comparison of sodium-ion battery performance with different B and N doped carbon samples

S.No	Material	Rate capacity ($mAhg^{-1}$)	Reference
1	B doped porous carbon	177 at 0.1 Ag^{-1}<br>109 at 0.5 Ag^{-1}	[6]
2	B doped porous carbon with a termite net structure (TNPBC)	200 at 0.1 Ag^{-1}<br>189 at 5Ag^{-1}	[7]
3	B and P doped carbon	221.9 at 0.05 Ag^{-1}<br>102 at 1 Ag^{-1}	[8]
4	B doped graphene quantum dots (B-GQD)	310 at 0.05 Ag^{-1}<br>126 at 1 Ag^{-1}	[9]
5	N-doped biomass carbon	152 at 0.05Ag^{-1}	[10]
6	N-doped carbon nanofiber	282 at 0.1Ag^{-1}<br>175 at 5 Ag^{-1}	[11]
7	CuS particles with nitrogen-doped carbon	308 at 0.1Ag^{-1}<br>237 at 5 Ag^{-1}	[12]
8	Nitrogen and oxygen co-doped porous carbon polyhedra	313 at 0.1 Ag^{-1}<br>and 228 at 1 Ag^{-1}	[13]
9	Boron doped Sb/SbO_2@rGO composites	346 at 0.05Ag^{-1}	[14]
10	Mesoporous boron carbon nitride (MBCN1)	349 at 0.05 Ag-1	Present work

Table S4: TPD (Temperature programmed desorption) data of MBCN samples

Sample	BET SA $m^2.g^{-1}$	Total Basicity (mmol/g)	
		Weak basic sites (30-250°C)	Strong basic sites (250-500°C)
MBCN1	1165.99	0.111	0.702
MBCN2	953.73	0.079	0.683
MBCN3	761.63	0.045	0.667
MBCN4	626.50	0.038	0.634

Supporting information references

[1] G. Kresse, D. Joubert, Physical Review B 1999, 59, 1758; G. Kresse, J. Furthmüller, Physical Review B 1996, 54, 11169.
[2] P. E. Blöchl, Physical Review B 1994, 50, 17953.
[3] J. P. Perdew, K. Burke, M. Ernzerhof, Phys Rev Lett 1996, 77, 3865.
[4] S. Grimme, J Comput Chem 2006, 27, 1787.
[5] K. Ramadass, C. I. Sathish, S. MariaRuban, G. Kothandam, S. Joseph, G. Singh, S. Kim, W. Cha, A. Karakoti, T. Belperio, J. B. Yi, A. Vinu, ACS Applied Materials & Interfaces 2020, 12, 11922; D.-H.

Park, K. S. Lakhi, K. Ramadass, M.-K. Kim, S. N. Talapaneni, S. Joseph, U. Ravon, K. Al-Bahily, A. Vinu, Chemistry – A European Journal 2017, 23, 10753; A. S. Jalilov, G. Ruan, C.-C. Hwang, D. E. Schipper, J. J. Tour, Y. Li, H. Fei, E. L. G. Samuel, J. M. Tour, ACS Applied Materials & Interfaces 2015, 7, 1376.
[6] H. F. Wang, C. Y. Fan, X. Y. Li, X. L. Wu, H. H. Li, H. Z. Sun, H. M. Xie, J. P. Zhang, C. Y. Tong, Electrochimica Acta 2017, 244, 86.
[7] D. Wang, Z. Y. Wang, Y. Li, K. Z. Dong, J. H. Shao, S. H. Luo, Y. G. Liu, X. W. Qi, Applied Surface Science 2019, 464, 422.
[8] K. Lu, S. Lu, T. Gu, X. Zheng, K. Ke, X. Li, R. Yang, Electrochemistry Communications 2019, 103, 22.
[9] A. P. Vijaya Kumar Saroja, M. S. Garapati, R. ShyiamalaDevi, M. Kamaraj, S. Ramaprabhu, Applied Surface Science 2020, 504, 144430.
[10] M. Khan, N. Ahmad, K. Lu, Z. Sun, C. Wei, X. Zheng, R. Yang, Solid State Ionics 2020, 346, 115223.
[11] Y. Lu, D. Li, C. Lyu, H. Liu, B. Liu, S. Lyu, T. Rosenau, D. Yang, Applied Surface Science 2019, 496, 143717.
[12] Y. Fang, X.-Y. Yu, X. W. Lou, Angewandte Chemie International Edition 2019, 58, 7744.
[13] Z. Li, L. Cai, K. Chu, S. Xu, G. Yao, L. Wei, F. Zheng, Dalton Transactions 2021, 50, 4335.
[14] J.-H. Liu, Y.-F. Li, Y.-H. Shi, J.-Z. Guo, J. Yang, X.-L. Wu, J.-P. Zhang, W. Hu, H.-Z. Sun, Journal of Physics D: Applied Physics 2021, 54, 315505.
